# Bone Marrow Adipose Tissue Is Not Required for Reconstitution of the Immune System following Irradiation in Male Mice

**DOI:** 10.3390/ijms25041980

**Published:** 2024-02-06

**Authors:** Jessica A. Keune, Carmen P. Wong, Adam J. Branscum, Scott A. Menn, Urszula T. Iwaniec, Russell T. Turner

**Affiliations:** 1 Skeletal Biology Laboratory, School of Nutrition and Public Health, Oregon State University, Corvallis, OR 97331, USA; 2 Biostatistics Program, School of Nutrition and Public Health, Oregon State University, Corvallis, OR 97331, USA; 3 Radiation Center, Oregon State University, Corvallis, OR 97331, USA; 4 Center for Healthy Aging Research, Oregon State University, Corvallis, OR 97331, USA

**Keywords:** bone marrow adipose tissue, BMAT, *Kit^W/W-v^*, bone architecture, microcomputed tomography, histology

## Abstract

Bone marrow adipose tissue (BMAT) is hypothesized to serve as an expandable/contractible fat depot which functions, in part, to minimize energy requirements for sustaining optimal hematopoiesis. We investigated whether BMAT is required for immune reconstitution following injury. Male wild type (WBB6F1, WT) and BMAT-deficient WBB6F1/J-*Kit^W^/Kit^W-v^*/J (*Kit^W/W-v^*) mice were lethally irradiated. Irradiation was followed by adoptive transfer of 1000 purified WT hematopoietic stem cells (HSCs). The extent of immune reconstitution in blood, bone marrow, and lymph nodes in the irradiated mice was determined using HSCs from green fluorescent protein (GFP)-expressing mice. We also evaluated skeletal response to treatment. Detection of GFP-positive B and T cells in peripheral blood at 4 and 9 weeks following adoptive transfer and in bone marrow and lymph nodes following necropsy revealed excellent immune reconstitution in both WT and BMAT-deficient mice. Adipocytes were numerous in the distal femur of WT mice but absent or rare in *Kit^W/W-v^* mice. Bone parameters, including length, mass, density, bone volume, microarchitecture, and turnover balance, exhibited few differences between WT and BMAT-deficient mice. The minimal differences suggest that BMAT is not required for reconstitution of the immune system following lethal radiation and is not a major contributor to the skeletal phenotypes of kit signaling-deficient mice.

## 1. Introduction

Bone marrow fat is an important depot for short- and long-duration storage of triglycerides. By middle age, bone marrow adipose tissue (BMAT) in humans typically contributes 5–10% of total body adipose tissue [1]. Fat depots generally act as dynamic energy reserves in which fatty acids are taken up by adipocytes from lipoproteins and subsequently released into the general circulation following lipolysis. Although BMAT can be highly labile [2], in contrast to white adipose tissue (WAT), bone marrow adipocytes often increase in number and sequester and esterify fatty acids during energy deprivation, implying that the bone marrow fat depot does not necessarily function as a traditional energy reservoir for peripheral tissues [3].

The physiological function of BMAT as well as its role in chronic disease initiation and progression are topics of current interest and debate [4,5,6,7,8,9,10,11,12,13]. We previously reviewed evidence that increased BMAT during fasting reflects an adaptive response to a negative energy balance [14]. An increase in BMAT and the resulting reduction in hematopoietic compartment size during long-duration fasting potentially promotes survival by lowering the energy expenditure required to maintain the turnover of hematopoietic cells. In support, age-related increases in BMAT and decreases in hematopoiesis are reversed by cold temperature stress, blood loss, and infection, conditions with increased requirement for hematopoiesis [15,16,17,18,19].

Mesenchymal lineage cells, including adipocytes, often express stem cell factor (kit ligand), the endogenous ligand for the protooncogene kit (CD117). Kit is a receptor tyrosine kinase expressed on early hematopoietic lineage cells and is required for their differentiation and survival [20]. In addition to regulating hematopoietic cell compartment size, it is possible that lipolysis of triglycerides stored in BMAT provides an energy source for hematopoietic cells. In this regard, Li et al. [21] concluded that BMAT is essential for recovery of the hematopoietic system and bone following exposure to an energy deficit, injury, or high-dose ionizing radiation.

Excessive quantities of BMAT may be undesirable and could contribute to skeletal pathologies, including bone loss associated with aging and gonadal insufficiency [22,23]. This possibility is supported by studies showing an inverse relationship between BMAT levels and bone formation as well as between BMAT levels and bone mass [24,25,26,27]. Osteoblasts and adipocytes are derived from mesenchymal stem cells residing in bone marrow, suggesting that an increase in BMAT may reflect a shift in the differentiation program of mesenchymal stem cells from osteoblasts to adipocytes [28].

Studies investigating the physiological role of BMAT in skeletal biology and its possible contribution to disease have been largely observational and causality has not been established. We have used mouse models with BMAT deficiency to address some of the limitations of prior research. These include two mouse strains (*Kit^Sl/Sld^* and *Kit^W/W-v^* having defects in expression of kit ligand and kit receptor, respectively), that do not accrue BMAT in their long bones or lumbar vertebrae [29]. Using female *Kit^W/W-v^* mice, we showed that the well-established increase in BMAT following ovariectomy had no effect on bone loss [30]. In contrast, BMAT deficiency in male *Kit^W/W-v^* mice was associated with enhanced bone loss during hindlimb unloading, suggesting BMAT had a protective action [31]. However, *Kit^W/W-v^* and *Kit^Sl/Sld^* mouse strains have bone marrow and skeletal abnormalities that could be due to BMAT-independent effects of kit signaling deficiency [30,31,32]. To address this limitation, we have refined the BMAT-deficient mouse models by restoring kit signaling to select tissues, including bone [29,33].

**Figure 1:**
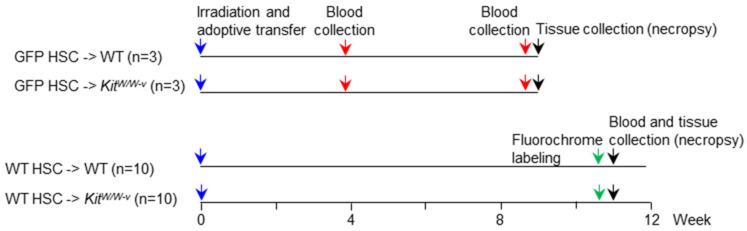
Experimental protocol: Mice were randomized into 4 groups: GFP HSC → WT (n = 3), GFP HSC → *Kit^W/W−v^* (n = 3), WT HSC → WT (n = 10), and WT HSC → *Kit^W/W−v^* (n = 10), lethally irradiated, and injected with purified HSCs from donor GFP or WT mice. GFP HSC → WT and GFP HSC → *Kit^W/W−v^* were maintained for 9 weeks with blood collected at 4 and 9 weeks post adoptive transfer. Mesenteric lymph nodes and bone marrow were collected at necropsy (2 days following 2nd blood collection) for assessment of GFP^+^ B and T cells. WT HSC → WT and WT HSC → *Kit^W/W−v^* were maintained for 11 weeks. Fluorochromes were administered 4 days and 1 day prior to necropsy in WT HSC → WT and WT HSC → *Kit^W/W−v^* mice to label mineralizing bone matrix. Humeri, 5th lumbar vertebrae, and femora were collected at necropsy for bone assessment using dual-energy absorptiometry (DXA; femur only), microcomputed tomography (μCT), and histomorphometry (femur only).

The objectives of this investigation were to assess the importance of BMAT following severe injury in (1) supporting hematopoiesis and (2) maintaining bone turnover balance. To accomplish the first objective, we examined the extent of immune reconstitution in lethally irradiated WT and BMAT-deficient *Kit^W/W-v^* mice after adoptive transfer of purified green fluorescent protein (GFP)-positive Kit^+^ hematopoietic stem cells (HSCs). To accomplish the second objective, we compared bone microarchitecture, turnover, and cell populations in WT and *Kit^W/W-v^* mice following irradiation and adoptive transfer of WT HSCs. The experimental protocol is outlined in Figure 1.

## 2. Results

### 2.1. Immune Cell Reconstitution

**Figure 2:**
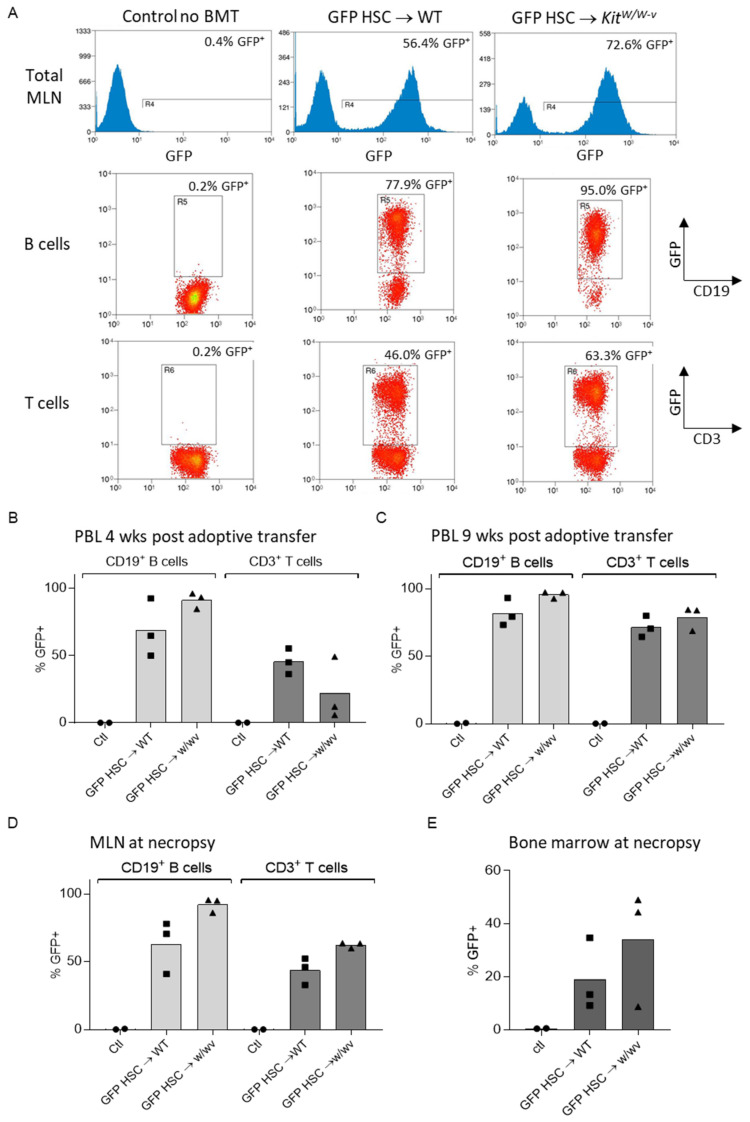
Immune cell reconstitution with donor HSC-derived GFP^+^ cells in irradiated recipient mice. (**A**) Representative FACS plots showing percentages of GFP^+^ cells of total mesenteric lymph node (MLN) cells, CD19^+^ B cells, and CD3^+^ T cells collected at necropsy 9 weeks following adoptive transfer. (**B**) Percentage of GFP^+^ B and T cells in peripheral blood lymphocytes (PBL) 4 weeks post irradiation/adoptive transfer. (**C**) Percentage of GFP^+^ B and T cells in PBL 9 weeks post irradiation/adoptive transfer. (**D**) Percentage of GFP^+^ B and T cells in MLN at necropsy. (**E**) Percentage of GFP^+^ bone marrow cells at necropsy.

The time course for immune cell reconstitution is shown in Figure 2. Representative FACS plots of mesenteric lymph node immune cells in respective treatment groups showed extensive reconstitution of B and T cell compartments with donor-derived GFP+ immune cells in recipient mice 9 weeks following adoptive transfer (Figure 2A). Reconstitution of recipient mouse B cells and T cells with donor HSC-derived GFP^+^ B cells and T cells was in progress in both WT and *Kit^W/W-v^* mice by 4 weeks post irradiation/adoptive transfer (Figure 2B). By 9 weeks, excellent reconstitution with donor-derived GFP^+^ B cells and T cells was observed in the blood (Figure 2C) and following necropsy in mesenteric lymph nodes (Figure 2D) in both WT and *Kit^W/W-v^* mice. Additionally, GFP^+^ cells were common in bone marrow (Figure 2E), indicating efficient repopulation of bone marrow from donor HSCs. Notably, the magnitude of hematopoietic cell reconstitution did not differ significantly between WT and *Kit^W/W-v^* mice. As expected, the control mice had no GFP-labeled cells.

### 2.2. Body Composition and Blood Glucose Levels

The effects of adoptive transfer of WT HSCs into irradiated WT and BMAT-deficient *Kit^W/W-v^* mice on total body and selected organ weights and on blood glucose levels at 11 weeks post irradiation/adoptive transfer are shown in Table 1. No significant differences in terminal body, spleen, or adrenal weights were noted and there was no difference in blood glucose levels at 11 weeks post irradiation/adoptive transfer. Abdominal WAT and seminal vesicle weights were lower in BMAT-deficient *Kit^W/W-v^* mice compared to WT mice.

### 2.3. Bone Microarchitecture

The effects of adoptive transfer of WT HSCs into WT and BMAT-deficient *Kit^W/W-v^* mice on humerus length, total humerus bone volume, cortical bone architecture in the midshaft humerus, and cancellous bone architecture in distal humerus epiphysis at 11 weeks post irradiation/adoptive transfer are shown in Table 2. No significant differences between the WT and BMAT-deficient *Kit^W/W-v^* mice were observed for humerus length, total humerus bone volume, or cortical (cross-sectional volume, cortical volume, marrow volume, polar moment of inertia) and cancellous (cancellous bone volume fraction, connectivity density, trabecular number, trabecular thickness, trabecular spacing) architecture.

The effects of irradiation/adoptive transfer of WT HSCs into WT and BMAT-deficient *Kit^W/W-v^* mice on total lumbar vertebra bone volume and cancellous bone architecture in the vertebral body are shown in Table 3. With the exception of trabecular thickness, which was lower in BMAT-deficient *Kit^W/W-v^* mice compared to WT mice, no group differences were noted for endpoints measured (total vertebral bone volume, cancellous bone volume fraction, connectivity density, trabecular number, trabecular thickness, trabecular spacing).

The effects of irradiation/adoptive transfer of WT HSCs into WT and BMAT-deficient *Kit^W/W-v^* mice on total femur bone mass and volume, on cortical bone architecture in midshaft femur, and cancellous bone architecture in distal femur metaphysis and epiphysis are shown in Table 4. No differences were observed for femur bone area, BMC, or BMD between WT and BMAT-deficient *Kit^W/W-v^* mice. Femur length, total bone volume, and cortical thickness likewise did not differ between the two groups. However, compared to WT mice, BMAT-deficient *Kit^W/W-v^* mice had lower cross-sectional, cortical, and marrow volume and lower polar moment of inertia. There were few differences in cancellous bone microarchitecture, the exceptions being that connectivity density in BMAT-deficient *Kit^W/W-v^* mice was higher than in WT mice in distal femur metaphysis but lower in distal femur epiphysis.

### 2.4. Static and Dynamic Histomorphometry

**Figure 3:**
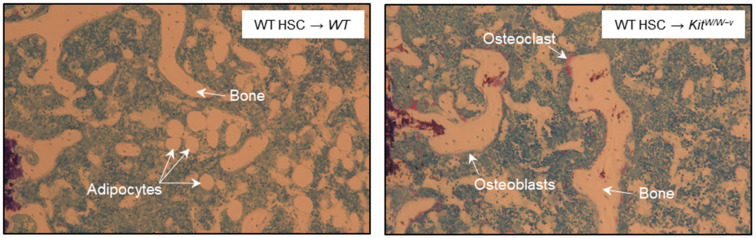
Representative histological images (10×) of distal femur metaphysis in region of interest of a WT HSC → WT mouse and a WT HSC → *Kit^W/W−v^* mouse. Please note the difference in BMAT between the 2 mice.

The effects of irradiation/adoptive transfer of WT HSCs into WT and BMAT-deficient *Kit^W/W-v^* mice on static and dynamic bone histomorphometry in distal femur metaphysis are shown in Table 5. No differences were observed for bone area fraction, mineralizing perimeter, mineral apposition rate, bone formation rates (perimeter, bone, and tissue referents), osteoclast-lined bone perimeter, or osteoblast-lined bone perimeter. Bone marrow adipocytes were common in WT mice but not detected in 8 out of 10 *Kit^W/W-v^* mice; adipocytes were present in very low numbers in the remaining 2 *Kit^W/W-v^* mice. Adipocyte area/tissue area and adipocyte density were much lower in BMAT-deficient *Kit^W/W-v^* mice than in WT mice but the few adipocytes that were present did not differ in size from those present in WT mice. The pronounced difference in adiposity can be appreciated in representative photomicrographs from a WT and a BMAT-deficient *Kit^W/W-v^* mouse (Figure 3).

## 3. Discussion

Adoptive transfer of 1000 purified WT HSCs was effective in reconstituting the immune systems of both WT and *Kit^W/W-v^* mice following lethal irradiation. In agreement with prior studies [31,33], adoptive transfer of WT HSCs did not result in accrual of BMAT in the femurs of *Kit^W/W-v^* mice. Furthermore, the near absence of BMAT did not prevent equalization of most bone parameters, particularly for cancellous bone, between WT and *Kit^W/W-v^* mice.

*Kit^W/W-v^* mice have a point mutation in the tyrosine kinase domain that inactivates the kit receptor in one allele and a mutation in the other allele that prevents translocation of the enzyme from the cytoplasm to the cell surface [34]. Consequently, activation of the kit receptor by a kit ligand is greatly reduced but sufficient to allow for survival. The kit ligand is produced in two forms, as membrane-bound and as soluble. The *Sl* mutation in *Kit^Sl/Sld^* mice deletes the entire *Sl* (kit ligand) gene whereas the Steel-Dickie (*Sl^d^*) mutation, resulting from an intragenic 4-kb deletion, removes sequences encoding transmembrane and cytoplasmic domains, limiting expression of the gene to produce only a soluble form of kit ligand [35].

Male *Kit^W/W-v^* mice, comparable in age (and housing conditions) to mice evaluated in the present study, had lower total body weight, WAT weight, and seminal vesicle weight and lower serum glucose levels compared to WT mice [31]. *Kit^W/W-v^* mice also had a lower cancellous bone volume fraction and higher bone turnover in the distal femur metaphysis [31]. Other studies described osteopenia associated with abnormalities in bone turnover balance in female *Kit^W/W-v^* mice [30] and male and female *Kit^Sl/Sld^* mice [32]. The differences between Kit signaling-deficient *Kit^W/W-v^* and *Kit^Sl/Sld^* mice and WT controls reported in the literature contrast with the minimal differences at cancellous bone sites reported in this study following the adoptive transfer of WT HSCs to normalize kit signaling. These findings suggest an important role for kit signaling in bone metabolism. However, they do not identify the underlying mechanisms or target cells.

We have administered soluble kit ligand to *Kit^Sl/Sld^* mice and have successfully adoptively transferred purified WT HSCs to male and female *Kit^W/W-v^* mice [29,31,33]. Neither approach resulted in the accrual of BMAT. However, a detailed comparison of the immune system and skeletal response of WT and *Kit^W/W-v^* mice to the adoptive transfer of WT HSCs had not been performed. In the present study, we confirmed that rescue of kit signaling in *Kit^W/W-v^* mice following adoptive transfer of WT HSCs does not result in the accrual of BMAT in the femur. On the other hand, normalization of kit signaling equalized many of the divergent bone parameters typically observed between WT and *Kit^W/W-v^* mice. This latter finding argues in favor of defective HSC differentiation as the primary cause for the skeletal abnormalities in kit signaling-deficient mice. However, the findings do not rule out a role for BMAT in supporting hematopoiesis.

There is strong in vitro evidence that adipocytes support the long-term survival of hematopoietic lineage cells via kit signaling [36] and a recent study concluded that lipolysis from BMAT is required to fuel bone cells and the marrow niche following energy deficits or injury [21]. However, weight loss due to a chronic energy deficit has highly variable effects on BMAT levels in humans as well as experimental animals [37,38]. Decreased weight due to involuntary caloric restriction often, but not always, leads to an increase in BMAT [37,39]. In contrast, weight loss resulting from increased hypothalamic leptin levels results in no change or decreased BMAT [40,41]. Additionally, the effects of injury induced by exposure to ionizing radiation on bone marrow adiposity are highly variable, ranging from no change to large increases [33,42]. This variability in response to irradiation may be due to radiation dose, skeletal site evaluated, or timing of measurement. The latter is likely important because high doses of ionizing radiation result in a rapid decrease in bone marrow cell density followed by gradual recovery [42]. Specifically, following a whole-body dose of ^137^Cs (6 Gy), bone marrow cell density declined by 77% after 1 day and 86% after 3 days. However, by 2 weeks post irradiation, recovery was well underway. Importantly, the effect of radiation on cells was not uniform; while total marrow cell numbers declined drastically post irradiation, osteoblast- and osteoclast-lined bone perimeter increased [42]. Furthermore, neither bone marrow collapse nor early-stage recovery (2 weeks) was accompanied by increased BMAT. In contrast, we observed increased BMAT following a longer duration (8 week) recovery post lethal irradiation [33]. Taken together, these findings suggest BMAT levels are a marker of recovery and argue against the hypothesis that energy stored in BMAT is critical to support bone marrow recovery following radiation-induced injury.

The present study directly tested the requirement for BMAT for reconstitution of the immune system following lethal (10 Gy) irradiation. Exposure to high-dose whole body radiation results in a large negative energy balance as well as massive cell death [42,43]. Despite the absence of BMAT, the reconstitution of lymphocyte cell populations in the present study with only 1000 donor GFP-expressing cells was similar in WT and BMAT-deficient *Kit^W/W-v^* mice. *Kit^W/W-v^* mice are mast cell-deficient, and we detected mast cells on bone surfaces in these mice following the adoptive transfer of WT HSCs, further supporting the successful restoration of normal hematopoietic cell differentiation. It is important to note that mesenchymal stem cells are radioresistant and, consequently, bone marrow cells expressing kit ligand are available to support hematopoiesis following irradiation. Taken together, these findings provide evidence that BMAT is not required for skeletal recovery from injury and energy deficit following exposure to high-dose radiation.

It is well established that kit is expressed by HSCs and kit ligand by differentiated mesenchymal cells, including fibroblasts, osteoblasts, and adipocytes [32]. However, recent studies suggest that there are kit-expressing populations of mesenchymal cells capable of differentiating into osteoblasts and adipocytes [44,45]. We previously demonstrated that the purified HSC population used for adoptive transfer can differentiate in vitro into osteoclasts but not osteoblasts or adipocytes [33]. We also demonstrated that inhibition of kit signaling with the receptor tyrosine kinase inhibitor gleevec rapidly reduced marrow adipocyte area and density in rats, suggesting that maintenance of mature adipocytes in bone marrow requires kit signaling [29]. Further research is necessary to determine whether the adoptive transfer of kit-expressing WT mesenchymal cells restores the ability of *Kit^W/W-v^* mice to accrue BMAT.

This study has limitations. The present study was performed in male mice. However, we have previously shown that female *Kit^W/W-v^* mice are also BMAT-deficient prior to and following the adoptive transfer of WT HSCs [33]. Lymphocytes can influence bone metabolism [46,47] and mast cells have been implicated in diet-induced obesity and diabetes in mice [48]. Additionally, mast cells participate in several skeletal disorders, including parathyroid bone disease [49]. Further research will be required to tease out the contribution of specific immune cells to the skeletal phenotypes of kit signaling-deficient mice. Although we did not include unirradiated WT and *Kit^W/W-v^* mice in the current analysis, the skeletal phenotypes of male and female *Kit^W/W-v^* mice and *Kit^S/Sld^* mice have been reported [30,31].

In summary, adoptive transfer of WT HSCs attenuated the skeletal differences between WT and BMAT-deficient *Kit^W/W-v^* mice, with the notable exception that mature adipocytes continued to be absent or very rare in the bone marrow of femurs of *Kit^W/W-v^* mice. The absence of BMAT had little or no influence on the reconstitution of immune, osteoblast, or osteoclast cell populations following lethal radiation. Taken together, these findings indicate that BMAT is not required for recovery from severe bone marrow injury. Our findings also support the conclusion that kit signaling plays an important role in bone turnover balance in mice. Finally, the present studies demonstrate the value of BMAT-deficient *Kit^W/W-v^* mice as a model for investigating the role of BMAT in bone physiology.

## 4. Materials and Methods

### 4.1. Animals

Four- to five-week-old male WBB6F1/J-*Kit^W^/Kit^W−v^*/J (*Kit^W/W−v^*) mice (n = 13) and their WT WBB6F1/J littermates (n = 13) were purchased from Jackson Laboratory (Bar Harbor, ME, USA) and used as adoptive transfer recipients. Additional WT and GFP-expressing (stock 006567 C57BL/6-Tg(CAG-EGFP)131Osb/LeySopJ) mice were purchased as the source of bone marrow and as HSC donors. The experimental protocol (ACUP 4304) was approved by the Institutional Animal Care and Use Committee at Oregon State University. The mice were single-housed at 32 °C for the duration of the study. Housing mice at 32 °C (near thermoneutral temperature) has been shown to minimize resting energy expenditure and prevent cold stress-induced reductions in peak bone mass and premature (while still growing) age-associated cancellous bone loss [50].

### 4.2. Experimental Protocol

The experimental protocol is outlined in Figure 1. At 3 weeks following arrival, the mice were randomized into 4 groups: (1) GFP HSC → *WT* (n = 3), (2) GFP HSC → *Kit^W/W−v^* (n = 3), (3) WT HSC → *WT* (n = 10), and (4) WT HSC → *Kit^W/W−v^* (n = 10), and lethally irradiated (two split doses of 5 Gy each, 10 Gy total; Gammacell 220 ^60^Co gamma irradiator). One day later, the mice were injected with purified HSCs from donor GFP or WT mice. Mice in groups 1 and 2 (GFP HSC → *WT* and GFP HSC → *Kit^W/W−v^*, respectively) were maintained for 9 weeks. Mice in groups 3 and 4 (WT HSC → *WT* and WT HSC → *Kit^W/W−v^*, respectively) were maintained for 11 weeks.

Purified WT and GFP HSCs were prepared as follows: whole bone marrow cells were harvested from the femora and tibia of 12 WT donor mice and 3 GFP donor mice, respectively. Lineage-negative (lin^−^) cells were enriched from bone marrow cells using magnetic cell separation with MACS lineage cell depletion kit (Miltenyi Biotec Inc., Auburn, CA, USA). Enriched lin^−^ bone marrow cells were incubated with anti-CD117 (c-kit) and anti-Sca-1 antibodies (eBioscience, San Diego, CA, USA). HSCs (Lin^−^Sca-1^+^ Kit^+^) were purified from enriched lin^−^ cells by flow cytometry and single-cell sorting using MoFlo XDP (Beckman Coulter, Indianapolis, IN, USA). Purified HSCs were resuspended in saline, and 200 μL containing 1000 donor HSCs was injected into the tail vein of each irradiated recipient mouse. The extent of cellular reconstitution of the hematopoietic compartment was determined by the presence of differentiated GFP^+^ immune cells in irradiated mice that received GFP HSC donor cells. GFP^+^ and GFP^-^ B and T cells in peripheral blood lymphocytes were measured by flow cytometry using B cell-specific (CD19) and T cell-specific (CD3) antibodies at 4 weeks and 9 weeks post adoptive transfer. Mesenteric lymph nodes and bone marrow were collected at necropsy (2 days following 2nd blood collection) and the percentage of GFP^+^ B and T cells in mesenteric lymph nodes, as well as bone marrow, were evaluated. Mice (n = 2) that did not undergo adoptive transfer were used as immunostaining controls.

Calcein (15 mg/kg) was administered by subcutaneous injection 4 days and 1 day prior to necropsy in group 3 and 4 mice (WT HSC → *WT* and WT HSC → *Kit^W/W−v^*, respectively) to label mineralizing bone matrix. For tissue collection, the mice were anesthetized with isoflurane anesthesia and then terminated by decapitation and exsanguination. Body weight (g), abdominal WAT weight (g), spleen weight (g), seminal vesicle weight (g), adrenal gland weight (g), and blood glucose (mg/dL) were recorded at necropsy. Humeri, 5th lumbar vertebrae, and femora were removed and placed in formalin for 24-h fixation, then stored at 4 °C in 70% ethanol prior to sequential analysis by dual-energy absorptiometry (DXA; femur only), microcomputed tomography (μCT), and histomorphometry (femur only).

### 4.3. Densitometry

Total femur bone area (cm^2^), bone mineral content (g), and bone mineral density (g/cm^2^) were measured using DXA (PIXImus 2; Lunar Corporation, Madison, WI, USA).

### 4.4. Micro-Computed Tomography

μCT was used for nondestructive three-dimensional evaluation of bone volume and architecture. Humeri, 5th lumbar vertebrae, and femora were scanned using a Scanco μCT40 scanner (Scanco Medical AG, Basserdorf, Switzerland) at a voxel size of 12 μm × 12 μm × 12 μm (55 kVp X-ray voltage, 145 μA intensity, and 200 ms integration time). The filtering parameters sigma and support were set to 0.8 and 1, respectively. The threshold value for evaluation was determined empirically and set at 245 (gray scale, 0–1000).

**Figure 4:**
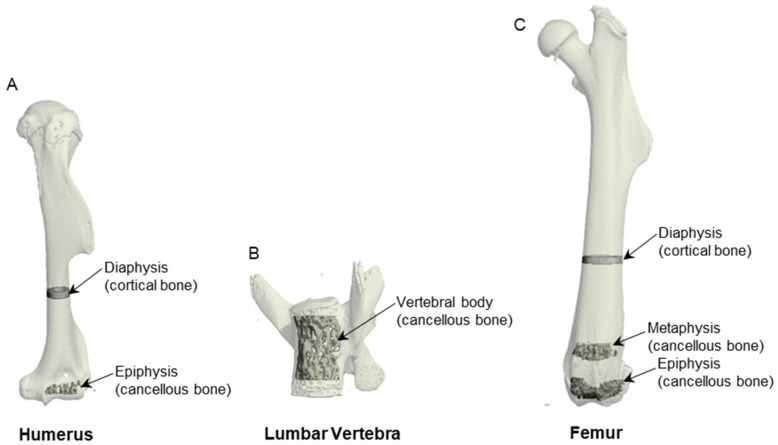
Regions of interest evaluated in (**A**) humerus, (**B**) lumbar vertebra, and (**C**) femur.

Cortical bone was evaluated in the humerus and femur diaphysis and cancellous bone was evaluated in the distal humerus epiphysis, vertebral body, and distal femur metaphysis and epiphysis (Figure 4). Assessment of cortical bone in the humerus and femur diaphysis began 60% distal to the top of the humeral/femoral head and consisted of 20 slices (240 µm) each. Automated contouring was used to delineate cortical bone from the marrow cavity. All cortical slices were visually examined for evidence of cancellous struts originating from the endocortex and manually removed when present. Direct cortical bone measurements included total cross-sectional volume (mm^3^), cortical volume (mm^3^), marrow volume (mm^3^), and cortical thickness (µm). Polar moment of inertia (I_polar_, mm^4^) was determined as a surrogate measure of bone strength in torsion.

Assessment of cancellous bone in the distal femur metaphysis began 45 slices (540 µm) proximal to the growth plate and included 40 slices (480 µm). The entire cancellous bone compartment was evaluated in the distal humerus epiphysis (23 ± 0.5 slices; 276 ± 6 µm), distal femur epiphysis (34 ± 0.5 slices; 408 ± 6 µm), and vertebral body (169 ± 1 slices; 2028 ± 12 µm). Direct cancellous bone measurements included bone volume fraction (bone volume/tissue volume; volume of total tissue occupied by cancellous bone, %), connectivity density (number of redundant connections per unit volume, 1/mm^3^), trabecular thickness (mean thickness of individual trabeculae, µm), trabecular number (number of trabecular intercepts per unit length, 1/mm), and trabecular spacing (distance between trabeculae, µm).

### 4.5. Histomorphometry

The histological methods used have been described [31]. In brief, distal femora were dehydrated in graded increases of ethanol and xylene, then embedded undecalcified in methyl methacrylate. Four µm thick sections were cut with a vertical bed microtome (Leica/Jung 2165) and fixed to slides with a dried precoated 1% gelatin solution. Unstained slides were used for measurements of fluorochrome labels. For cell-based measurements, slides were stained with tartrate-resistant acid phosphatase and counterstained with toluidine blue (Sigma, St. Louis, MO, USA). All data were collected using the OsteoMeasure System (OsteoMetrics, Inc., Atlanta, GA, USA).

The sampling site for the distal femur metaphysis was located 0.25–1.25 mm proximal to the growth plate. Dynamic (fluorochrome) histological measurements included mineralizing perimeter (mineralizing perimeter/bone perimeter; %), mineral apposition rate (distance between two fluorochrome markers that comprise a double label divided by the 3-day label interval; μm/day), and bone formation rate (mineralizing perimeter multiplied by mineral apposition rate normalized to bone perimeter; μm^2^/μm/year, bone area; %/year, and tissue area; %/year).

Static (cell-based) histological measurements include bone area fraction (bone area/tissue area, %), osteoclast perimeter (osteoclast perimeter/bone perimeter; %), osteoblast perimeter (osteoblast perimeter/bone perimeter; %), bone marrow adiposity (adipocyte area/tissue area; %), adipocyte density (#/mm^2^), and adipocyte size (μm^2^). Osteoclast perimeter was determined as a percentage of cancellous bone perimeter covered by multinucleated cells with an acid phosphatase-positive (stained red) cytoplasm. Osteoblast perimeter was determined as a percentage of total bone perimeter lined by plump cuboidal cells located immediately adjacent to a layer of osteoid in direct physical contact with bone. Adipocytes were identified as large circular or oval-shaped cells bordered by a prominent cell membrane lacking cytoplasmic staining due to alcohol extraction of intracellular lipids during processing.

### 4.6. Statistical Analysis

Mean outcomes for WT and BMAT-deficient *Kit^W/W-v^* mice were compared using Welch’s two-sample *t*-test or the distribution-free Wilcoxon–Mann–Whitney test (when normality was violated). Residual analysis and Levene’s test were used to assess normality and homogeneity of variance. The Benjamini and Hochberg [51] method for maintaining the false discovery rate at 5% was used to adjust for multiple comparisons. Differences were considered significant at *p* ≤ 0.05. All data are presented as mean ± SE. Data analysis was performed using R version 4.1.2.

## Figures and Tables

**Table 1:** Effects of adoptive transfer of WT HSCs into irradiated WT and BMAT-deficient *Kit^W/W-v^* mice on total body weight, abdominal white adipose tissue weight, spleen weight, seminal vesicle weight, adrenal weight, and blood glucose levels at 11 weeks post irradiation/adoptive transfer.

	WT HSC → WT	WT HSC → *Kit^W/W-v^*
Body weight (g)	26.6 ± 0.5	24.3 ± 0.6
Abdominal white adipose tissue (g)	0.79 ± 0.07	0.50 ± 0.05 ^a^
Spleen weight (g)	0.059 ± 0.002	0.057 ± 0.003
Seminal vesicle weight (g)	0.197 ± 0.013	0.145 ± 0.009 ^a^
Adrenal weight (g)	0.005 ± 0.000	0.006 ± 0.001
Blood glucose (mg/dL)	143 ± 8	137 ± 10

Data are mean ± SE, n = 10/group; ^a^ Different from WT HSC → WT, *p* ≤ 0.05.

**Table 2:** Effects of adoptive transfer of WT HSCs into irradiated WT and BMAT-deficient *Kit^W/W-v^* mice on humerus length, total humerus bone volume, cortical bone architecture in the midshaft humerus, and cancellous bone architecture in distal humerus epiphysis at 11 weeks post irradiation/adoptive transfer.

	WT HSC → WT	WT HSC → *Kit^W/W-v^*
Total humerus		
Length (mm)	12.5 ± 0.1	12.7 ± 0.1
Bone volume (mm^3^)	10.0 ± 0.2	9.6 ± 0.1
Midshaft humerus (cortical bone)		
Cross-sectional volume (mm^3^)	0.19 ± 0.00	0.18 ± 0.00
Cortical volume (mm^3^)	0.13 ± 0.00	0.12 ± 0.00
Marrow volume (mm^3^)	0.06 ± 0.00	0.06 ± 0.00
Cortical thickness (µm)	219 ± 3	211 ± 1
I_polar_ (mm^4^)	0.09 ± 0.00	0.08 ± 0.00
Distal humerus epiphysis (cancellous bone)		
Bone volume/tissue volume (%)	33.9 ± 0.9	32.3 ± 1.0
Connectivity density (1/mm^3^)	115.9 ± 8.3	116.6 ± 11.5
Trabecular number (1/mm)	9.0 ± 0.6	8.2 ± 0.4
Trabecular thickness (µm)	64 ± 1	63 ± 1
Trabecular spacing (µm)	123 ± 3	130 ± 5

Data are mean ± SE, n = 10/group.

**Table 3:** Effects of adoptive transfer of WT HSCs into irradiated WT and BMAT-deficient *Kit^W/W-v^* mice on total lumbar vertebra bone volume and cancellous bone architecture in the vertebral body at 11 weeks post irradiation/adoptive transfer.

	WT HSC → WT	WT HSC → *Kit^W/W-v^*
Total lumbar vertebra		
Bone volume (mm^3^)	5.1 ± 0.1	5.0 ± 0.1
Vertebral body (cancellous bone)		
Bone volume/tissue volume (%)	18.2 ± 0.3	17.2 ± 0.5
Connectivity density (1/mm^3^)	118.7 ± 4.6	124.7 ± 5.9
Trabecular number (1/mm)	4.2 ± 0.1	4.2 ± 0.1
Trabecular thickness (µm)	48 ± 1	46 ± 1 ^a^
Trabecular spacing (µm)	230 ± 3	236 ± 3

Data are mean ± SE, n = 10/group; ^a^ Different from WT HSC → WT, *p* ≤ 0.05.

**Table 4:** Effects of adoptive transfer of WT HSCs into irradiated WT and BMAT-deficient *Kit^W/W-v^* mice on total femur bone mass (densitometry) and total femur bone volume, cortical bone architecture in the midshaft femur, and cancellous bone architecture in distal femur metaphysis and epiphysis (µCT) at 11 weeks post irradiation/adoptive transfer.

	WT HSC → WT	WT HSC → *Kit^W/W-v^*
Densitometry		
Bone area (cm^2^)	0.44 ± 0.00	0.42 ± 0.01
BMC (g)	0.022 ± 0.000	0.020 ± 0.000
BMD (g/cm^2^)	0.050 ± 0.001	0.048 ± 0.001
microComputed Tomography		
Total femur		
Length (mm)	15.3 ± 0.1	15.4 ± 0.1
Bone volume (mm^3^)	18.98 ± 0.29	18.44 ± 0.30
Midshaft femur (cortical bone)		
Cross-sectional volume (mm^3^)	0.35 ± 0.00	0.32 ± 0.01 ^a^
Cortical volume (mm^3^)	0.19 ± 0.00	0.18 ± 0.002 ^a^
Marrow volume (mm^3^)	0.16 ± 0.00	0.14 ± 0.004 ^a^
Cortical thickness (µm)	210 ± 2	213 ± 2
I_polar_ (mm^4^)	0.27 ± 0.01	0.23 ± 0.01 ^a^
Distal femur metaphysis (cancellous bone)		
Bone volume/tissue volume (%)	8.5 ± 0.6	10.0 ± 0.7
Connectivity density (1/mm^3^)	55.0 ± 4.3	93.0 ± 9.3 ^a^
Trabecular number (1/mm)	4.1 ± 0.1	4.4 ± 0.1
Trabecular thickness (µm)	47 ± 1	45 ± 1
Trabecular spacing (µm)	253 ± 5	239 ± 8
Distal femur epiphysis (cancellous bone)		
Bone volume/tissue volume (%)	26.0 ± 0.5	26.3 ± 0.5
Connectivity density (1/mm^3^)	141.1 ± 5.2	114.0 ± 5.3 ^a^
Trabecular number (1/mm)	5.5 ± 0.1	5.4 ± 0.1
Trabecular thickness (µm)	57 ± 0	58 ± 1
Trabecular spacing (µm)	180 ± 4	190 ± 4

Data are mean ± SE, n = 10/group; ^a^ Different from WT HSC → WT, *p* ≤ 0.05.

**Table 5:** Effects of adoptive transfer of WT HSCs into irradiated WT and BMAT-deficient *Kit^W/W-v^* mice on dynamic and static bone histomorphometry in distal femur metaphysis at 11 weeks post irradiation/adoptive transfer.

	WT HSC → WT	WT HSC → *Kit^W/W-v^*
Bone area/tissue area (%)	8.2 ± 0.5	8.7 ± 1.2
Mineralizing perimeter/bone perimeter (%)	6.5 ± 1.0	6.0 ± 1.4
Mineral apposition rate (µm/d)	1.07 ± 0.10	1.16 ± 0.08
Bone formation rate/bone perimeter (μm^3^/μm^2^/y)	27.0 ± 5.8	28.2 ± 8.3
Bone formation rate/bone volume (%/y)	144.1 ± 31.5	141.6 ± 36.5
Bone formation rate/tissue volume (%/y)	11.8 ± 2.8	13.7 ± 4.2
Osteoclast perimeter/bone perimeter (%)	6.5 ± 1.5	6.1 ± 1.6
Osteoblast perimeter/bone perimeter (%)	7.7 ± 1.5	11.1 ± 2.1
Adipocyte area/tissue area (%)	8.9 ± 1.1	0.2 ± 0.1 ^a^
Adipocyte density (#/mm^2^)	92.5 ± 10.0	1.8 ± 1.2 ^a^
Adipocyte size (µm^2^)	943 ± 34	927 ± 334 *

Data are mean ± SE, n = 10/group; * n = 2 *Kit^W/W-v^* mice with BMAT; ^a^ Different from WT HSC → WT, *p* ≤ 0.05.

## Data Availability

The data will be made available to anyone upon request.
